# Cardiovascular composite events and the healthy worker effect among noise-exposed workers: a retrospective cohort study

**DOI:** 10.3389/fpubh.2026.1889187

**Published:** 2026-07-07

**Authors:** Jiahui Liu, Qi Li, Jiamin Liu

**Affiliations:** 1 Hunan Prevention and Treatment Institute for Occupational Diseases, Affiliated Prevention and Treatment Institute for Occupational Diseases of University of South China, Changsha, China; 2 The First Affiliated Hospital of Guangzhou Medical University (Loudi Hospital), Loudi, China

**Keywords:** cardiovascular disease, Cox regression, healthy worker effect, occupational noise exposure, retrospective cohort study

## Abstract

**Background:**

Occupational noise exposure is prevalent, but its cardiovascular effects are under recognized. Most studies are cross-sectional, focus on single endpoints, and rarely account for the healthy worker effect (HWE). This study aimed to identify risk factors for cardiovascular composite events in noise-exposed workers and further assess this effect.

**Methods:**

This retrospective cohort study enrolled 1,381 noise-exposed workers with normal blood pressure and normal electrocardiogram at baseline in 2020. All participants were followed annually through 2023. The primary endpoint was a composite cardiovascular event (hypertension or electrocardiographic abnormality). Cox proportional hazards models were used to identify associated factors. Restriction analysis, restricted cubic spline analysis, and sensitivity analyses were performed to assess robustness.

**Results:**

Multivariable Cox regression identified systolic blood pressure (SBP) (HR = 1.012, 95% CI 1.005–1.020, *p* < 0.001) and smoking (HR = 1.191, 95% CI 1.029–1.377, *p* = 0.019) as independent risk factors. Work duration >120 months showed an apparent protective effect (HR = 0.621, 95% CI 0.466–0.826, *p* = 0.001). After excluding pre-employment workers, the reduced risk for >120 months persisted (HR = 0.787, 95% CI 0.638–0.969, *p* = 0.024). Forcing age into the multivariable model did not materially change the effect estimates. Sensitivity analysis showed that baseline SBP ≥ 130 mmHg was associated with a 66.3% increased risk of composite events (HR = 1.663, 95% CI 1.327–2.085, *p* < 0.001). For the hypertension endpoint, combined exposure to noise and dust (HR = 1.896, 95% CI 1.219–2.949, *p* = 0.005) and combined exposure to noise and chemical agents (HR = 1.580, 95% CI 1.013–2.463, *p* = 0.044) significantly increased risk.

**Conclusion:**

Smoking and elevated SBP are independent risk factors for cardiovascular composite events in noise-exposed workers. The apparent protective effect of work duration is consistent with the HWE. Combined exposure to noise with dust or chemical agents significantly increases the risk of hypertension.

## 1 Introduction

Occupational noise is widespread in metal processing, manufacturing, construction, transportation, and other industries. Globally, hundreds of millions of workers are exposed to occupational noise during their work activities (1). A meta-analysis involving 157,370 workers indicated that the prevalence of occupational noise exposure is 17% (2). As the world’s largest industrial country, China faces a substantial burden of occupational noise hazards. National surveillance data from 2020 showed that the rate of workplace noise exceeding permissible levels in the manufacturing industry was high, particularly in small and medium-sized enterprises (3). Surveillance in Jiangsu Province in 2022 similarly indicated that noise remained the most prevalent physical hazard in workplaces (4). Meanwhile, the Summary of the China Cardiovascular Health and Disease Report 2024 identified cardiovascular disease as the leading cause of death among urban and rural residents in China (5). This highlights the cardiovascular effects of occupational noise exposure as a critical public health issue.

From a biological perspective, long-term noise exposure activates the autonomic nervous system and the hypothalamic–pituitary–adrenal axis. This promotes the release of catecholamines and cortisol, which in turn induces oxidative stress, endothelial dysfunction, and vascular inflammation (6). Epidemiological evidence has consistently linked occupational noise exposure to adverse cardiovascular outcomes. A meta-analysis by Skogstad et al., including 12 prospective studies, reported that occupational noise exposure significantly increased the risk of hypertension (HR = 1.68, 95% CI 1.10–2.57) and cardiovascular disease (RR = 1.34, 95% CI 1.15–1.56) (7). A meta-analysis of Chinese noise-exposed workers further reported that noise exposure was associated with a 2.27–fold increased risk of electrocardiographic abnormality (95% CI 1.96–2.62) (8). A Swedish soft paper mill cohort study found that noise >90 dBA was associated with increased ischemic heart disease mortality (SMR = 1.27), and that combined exposure to noise and paper dust further elevated ischemic heart disease mortality (SMR 1.39, 95% CI 1.15–1.67) (9).

These studies have provided preliminary evidence for the cardiovascular hazards of occupational noise. However, compared with environmental noise, the cardiovascular effects of occupational noise have received relatively limited attention. Most existing studies are cross-sectional in design, and longitudinal cohort evidence remains insufficient. The majority have focused on hypertension as a single endpoint, and cohort studies using electrocardiographic abnormality as a longitudinal outcome are even rarer. Electrocardiographic abnormalities may appear before a significant rise in blood pressure, suggesting their potential as early indicators of noise-induced subclinical cardiac electrophysiological changes. Furthermore, the HWE, commonly present in occupational cohorts, can create a spurious inverse association between work duration and health outcomes (10), yet most studies have not adequately addressed this bias in their analyses (11). Therefore, this study used a 3-year follow-up retrospective cohort of noise-exposed workers, with hypertension or electrocardiographic abnormality as the composite cardiovascular endpoint. The objectives were to identify independent risk factors, to assess the healthy worker effect (HWE), and to explore the synergistic impact of mixed occupational exposures on cardiovascular health.

## 2 Materials and methods

### 2.1 Study design and population

Inclusion criteria were: (1) age ≥18 years; (2) noise-exposed workers who underwent occupational health examination at the Health Management Center of Hunan Prevention and Treatment Institute for Occupational Diseases in 2020, with both blood pressure and electrocardiogram normal at baseline. Exclusion criteria were: (1) previous diagnosis of hypertension or electrocardiographic abnormality; (2) incomplete physical examination data; (3) malignant tumors, severe hematologic diseases, or severe hepatic or renal insufficiency; (4) inability to complete questionnaires and physical examinations due to communication barriers or cognitive impairment. In 2020, 1,381 noise-exposed workers with normal blood pressure and normal electrocardiogram at baseline and complete occupational health examination records for four consecutive years (2020–2023) were enrolled. Workers with 0 months of work duration were defined as pre-employment workers–those who had completed pre-placement medical examination but had not yet started work or had just started work at the time of baseline examination. This study was approved by the Ethics Committee of Hunan Prevention and Treatment Institute for Occupational Diseases (Approval No. 2025091001–KY).

### 2.2 Outcome definition

The primary endpoint was a composite cardiovascular event. This was defined as the first occurrence of hypertension or electrocardiographic abnormality during the follow-up period (2021–2023). Hypertension was defined as systolic blood pressure (SBP) ≥ 140 mmHg and/or diastolic blood pressure (DBP) ≥ 90 mmHg. Blood pressure was measured by trained nurses using calibrated electronic sphygmomanometers. Participants rested seated for ≥5 min before measurement, and the right upper arm blood pressure was recorded. Electrocardiographic abnormalities included sinus tachycardia, sinus bradycardia, sinus arrhythmia, ST-segment changes, conduction block, T-wave changes, and left ventricular high voltage, among other abnormalities. Two certified physicians independently interpreted the electrocardiograms (ECGs). Discrepancies were resolved by consensus. Recognizing the clinical heterogeneity of electrocardiographic abnormalities, hypertension and electrocardiographic abnormality were also analyzed separately as secondary endpoints. Survival time was defined as the number of years from the baseline examination in 2020 to the first occurrence of the endpoint (1, 2, or 3 years). Those who did not develop an endpoint were censored at year 3.

### 2.3 Exposures and covariates

The following data were collected: demographic characteristics including age and sex; occupational exposures including work duration (categorized as 0 months [pre-employment], 1–60 months, 61–120 months, and >120 months) and hazard exposure groups (noise only, noise + dust, noise + chemical agents, noise + heat/other); lifestyle factors including smoking, alcohol consumption, and family history; clinical indicators including baseline SBP, diastolic blood pressure, and heart rate; audiometric indicators including bilateral high-frequency hearing threshold average (BHFTA) and pure-tone average (PTA); and routine blood parameters including hemoglobin, white blood cell count, and platelet count.

Smoking was defined as currently smoking ≥1 cigarette per day for ≥6 months. Alcohol consumption was defined as drinking alcohol ≥1 time per week for ≥6 months. A family history of cardiovascular disease was defined as a history of hypertension or coronary heart disease in a first-degree relative. Audiometric testing was performed in a soundproof booth using pure-tone audiometry according to the national standard (GB/T 7583). BHFTA was calculated as the average hearing threshold at 3000, 4000, and 6,000 Hz across both ears. PTA was calculated as the average hearing threshold at 500, 1000, and 2000 Hz across both ears. All examinations were conducted in accordance with the Technical Specifications for Occupational Health Surveillance (GBZ 188–2014). Workers classified as “noise-exposed” met the L_eq, 8 h ≥ 80 dB(A) standard per workplace monitoring (GBZ 188–2014); individual dosimetry data were not retained for this retrospective study.

### 2.4 Statistical analysis

All analyses were performed using R version 4.5.3. Baseline characteristics were compared between workers who developed a composite event and those who did not. Continuous variables were presented as mean ± standard deviation and were compared using independent two-sample t-tests. Categorical variables were presented as frequency (percentage) and were compared using chi-square tests. Cox proportional hazards regression models were used to identify factors associated with the composite cardiovascular event. Variables with *p* < 0.1 in univariable analysis were entered into the multivariable Cox model. For multicategorical variables, the first level served as the reference (work duration: 0 months; hazard group: noise only). Collinearity was assessed using variance inflation factors (VIF) for all continuous variables in each multivariable model; all VIF values were below 3, indicating no significant multicollinearity. The proportional hazards assumption was tested using Schoenfeld residuals.

The healthy worker effect was evaluated using three complementary approaches. First, restriction was performed: pre-employment workers (0 months) were excluded, and the multivariable Cox regression was repeated among active workers to observe changes in the HRs for work duration categories. Second, the dose–response relationship between work duration and the composite endpoint was explored by modeling work duration as a continuous variable (months) in a separate Cox model with adjustment for baseline SBP, smoking, platelet count, sex, and PTA; restricted cubic splines were fitted, and nonlinearity was tested using the Wald χ^2^ test. Third, age was forced into the multivariable model for the composite endpoint to rule out residual confounding by age.

Several sensitivity analyses were conducted. Baseline SBP was dichotomized (≥130 mmHg vs. <130 mmHg) and the multivariable Cox model was refitted. A semi-quantitative cumulative exposure index was constructed by multiplying work duration (months) by an exposure intensity weight (noise only = 1, noise + dust/chemical agents = 1.5, noise + heat/other = 1.2) and substituted for the categorical work duration variable in the composite endpoint model. E-value analyses were performed to quantify the potential impact of unmeasured confounding on the main findings. Secondary endpoint analyses used hypertension and electrocardiographic abnormality as dependent variables in separate multivariable Cox models. All tests were two-sided, and *p* < 0.05 was considered statistically significant.

## 3 Results

### 3.1 Baseline characteristics

A total of 1,381 workers were enrolled. Among them, 606 did not develop a composite event and 775 developed a composite event during follow-up. Compared with the non-event group, the event group had significantly higher baseline SBP, DBP, PTA, and platelet count (all *p* < 0.05). The distribution of work duration and the proportion of smokers also differed significantly between the two groups (both *p* < 0.05). The event group had a higher proportion of pre-employment workers (0 months) and a lower proportion of workers with work duration exceeding 120 months. There were no significant differences in age, heart rate, BHFTA, hemoglobin, white blood cell count, sex, hazard exposure group, family history, or alcohol consumption between the two groups (all *p* > 0.05). Detailed data are presented in Supplementary Table S1.

### 3.2 Multivariable Cox regression for the composite endpoint

Collinearity diagnostics showed high correlation between DBP and SBP (*r* = 0.75). Only SBP was retained in subsequent analyses. Variables with *p* < 0.1 in univariable analysis were entered into the multivariable Cox model. Multivariable Cox regression showed that each 1 mmHg increase in SBP was associated with a 1.2% increase in the risk of the composite endpoint (HR = 1.012, 95% CI 1.005–1.020, *p* < 0.001). Compared with 0 months of work duration, the HRs for 1–60 months and 61–120 months showed a decreasing trend, and the risk was significantly reduced by 38% for >120 months (HR = 0.621, 95% CI 0.466–0.826, *p* = 0.001). Smoking was associated with a 19% increased risk (HR = 1.191, 95% CI 1.029–1.377, *p* = 0.019). Platelet count and PTA showed marginal associations (*p* = 0.080 and *p* = 0.082, respectively). Sex showed no significant association. The proportional hazards assumption was satisfied (global *p* = 0.806) (Table 1).

**Table 1:** Multivariable Cox regression analysis for the composite endpoint.

Variable	HR	95% CI	***p***
SBP (mmHg)	1.012	1.005–1.020	<0.001
Platelet count (×10^9^/L)	1.001	1.000–1.002	0.080
PTA (dB HL)	1.016	0.998–1.034	0.082
Work duration			
0 months	1.000	Reference	
1–60 months	0.785	0.613–1.004	0.054
61–120 months	0.788	0.608–1.022	0.072
>120 months	0.621	0.466–0.826	0.001
Smoking			
No	1.000	Reference	
Yes	1.191	1.029–1.377	0.019
Sex			
Female	1.000	Reference	
Male	1.208	0.860–1.689	0.276

The model included all variables listed in the table. SBP: systolic blood pressure; PTA: binaural pure-tone average (speech frequency).

### 3.3 Multivariable analysis for the hypertension endpoint

The same variable selection and modeling strategy was applied. Because age violated the proportional hazards assumption, an age-stratified model was used (quartiles: ≤27, 28–31, 32–37, ≥38 years). Age HRs were therefore not estimated. Each 1 mmHg increase in SBP was associated with an 8.5% increase in the risk of the hypertension endpoint (HR = 1.085, 95% CI 1.064–1.099, *p* < 0.001). Compared with noise exposure alone, combined exposure to noise and dust (HR = 1.896, 95% CI 1.219–2.949, *p* = 0.005) and combined exposure to noise and chemical agents (HR = 1.580, 95% CI 1.013–2.463, *p* = 0.044) significantly increased the risk of the hypertension endpoint. Each 1 g/L increase in hemoglobin was associated with a 1.7% increase in risk (HR = 1.017, 95% CI 1.002–1.032, *p* = 0.031). The model PH assumption was satisfied (global *p* = 0.624) (Table 2).

**Table 2:** Multivariable Cox regression analysis for the hypertension endpoint (age-stratified).

Variable	HR	95% CI	***p***
SBP (mmHg)	1.085	1.064–1.099	<0.001
BHFTA (dB HL)	1.009	0.996–1.023	0.173
Hemoglobin (g/L)	1.017	1.002–1.032	0.031
White blood cell count (×10^9^/L)	1.017	0.936–1.105	0.693
Heart rate (beats/min)	1.005	0.983–1.029	0.651
Hazard factor category			
Noise only	1.000	Reference	
Noise + dust	1.896	1.219–2.949	0.005
Noise + chemical agents	1.580	1.013–2.463	0.044
Noise + heat/others	1.284	0.651–2.532	0.470
Sex			
Female	1.000	Reference	
Male	3.077	0.735–12.878	0.124
Family history			
No	1.000	Reference	
Yes	1.281	0.846–1.938	0.241

Because age violated the proportional hazards assumption, the model was stratified by age (quartiles: ≤27, 28–31, 32–37, ≥38 years). Age HRs were therefore not estimated. BHFTA: binaural high-frequency average hearing threshold. The model included all variables listed in the table.

### 3.4 Multivariable analysis for the electrocardiographic abnormality endpoint

The results of the multivariable Cox model for electrocardiographic abnormality are shown in Table 3. Each 1-year increase in age was associated with a 2% lower risk of the endpoint (HR = 0.984, 95% CI 0.972–0.995, *p* = 0.007). Work duration >120 months showed a marginal inverse association (HR = 0.728, 95% CI 0.517–1.026, *p* = 0.070). Smoking was associated with a 31.3% increased risk (HR = 1.313, 95% CI 1.126–1.531, *p* < 0.001). Higher PTA was associated with increased risk (HR = 1.048, 95% CI 1.025–1.072, *p* < 0.001), whereas higher BHFTA was associated with decreased risk (HR = 0.983, 95% CI 0.973–0.994, *p* = 0.002). Hazard exposure groups and platelet count were not significantly associated with the endpoint. The proportional hazards assumption was satisfied (global *p* = 0.744).

**Table 3:** Multivariable Cox regression analysis for the electrocardiographic abnormality endpoint.

Variable	HR	95% CI	***p***
Age (years)	0.984	0.972–0.995	0.007
PTA (dB HL)	1.048	1.025–1.072	<0.001
BHFTA (dB HL)	0.983	0.973–0.994	0.002
Platelet count (×10^9^/L)	1.001	1.000–1.002	0.198
Work duration			
0 months	1.000	Reference	
1–60 months	0.807	0.621–1.049	0.109
61–120 months	0.811	0.612–1.074	0.144
>120 months	0.728	0.517–1.026	0.070
Smoking			
No	1.000	Reference	
Yes	1.313	1.126–1.531	<0.001
Hazard factor category			
Noise only	1.000	Reference	
Noise + dust	0.824	0.673–1.009	0.061
Noise + chemical agents	0.977	0.807–1.184	0.813
Noise + heat/others	1.122	0.830–1.516	0.454

All VIF<2; Pearson r between PTA and BHFTA = 0.50.

### 3.5 Subgroup analysis: restriction of pre-employment workers

To evaluate the HWE through restriction, multivariable Cox regression was repeated among active workers (*n* = 1,264) after excluding pre-employment workers (0 months) (Table 4). In the total population, with 0 months as the reference, >120 months was associated with a 37.9% lower risk (HR = 0.621, 95% CI 0.466–0.826, *p* = 0.001). In the active worker population, with 1–60 months as the reference, the risk for 61–120 months was similar (HR = 0.999, 95% CI 0.843–1.185, *p* = 0.099), and the risk for >120 months remained reduced by 21.3% (HR = 0.787, 95% CI 0.638–0.969, *p* = 0.024).

**Table 4:** Restriction of pre-employment workers on the composite endpoint.

Work duration	Overall (n=1381)	Active workers (n=1264)
0 months	Reference	/
1–60 months	0.785 (0.613–1.004) *p*=0.054	Reference
61–120 months	0.788 (0.608–1.022) *p*=0.072	0.999 (0.843–1.185) *p*=0.099
>120 months	0.621 (0.466–0.826) *p*=0.001	0.787 (0.638–0.969) *p*=0.024

Data are presented as HR (95% CI). Active workers are defined as workers with duration ≥1 month. HRs were derived from multivariable Cox regression models adjusted for systolic blood pressure, smoking, platelet count, sex, and PTA.

Forest plots for the subgroup analysis of smoking and SBP are presented in Supplementary Figure S1. The effects of smoking and SBP were robust across the overall, male, and active worker subgroups (consistent HR direction, all *p* < 0.05). The smoking effect in the female subgroup could not be estimated due to the small sample size.

### 3.6 Restricted cubic spline analysis

**Figure 1:**
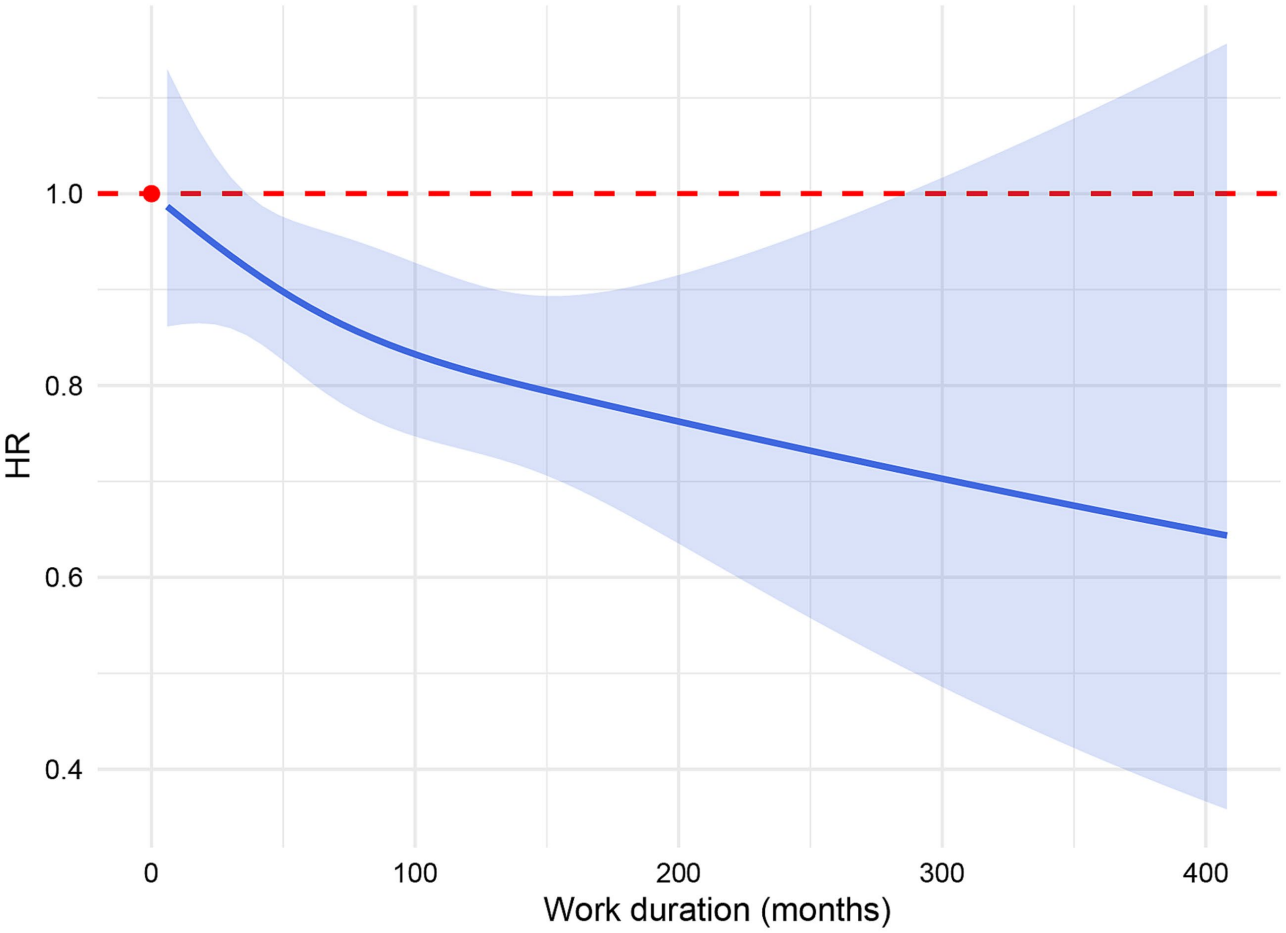
Dose–response relationship between work duration and the composite endpoint (restricted cubic spline). Knots were placed at the 10th, 50th, and 90th percentiles, with a step size of 6 months. Work duration of 0 months served as the reference. The model was adjusted for systolic blood pressure (112 mmHg), smoking (no), platelet count (228 × 10^9^/L), sex (male), and pure-tone average (PTA = 17.3 dB HL). The solid line represents the hazard ratio (HR), the shaded area indicates the 95% confidence interval, and the red dashed line marks HR = 1.

Restricted cubic spline analysis was performed to examine the dose–response relationship between work duration and the composite endpoint. The model was adjusted for SBP, smoking, platelet count, sex, and PTA. The overall association of work duration was marginally significant (*χ^2^* = 5.30, df = 2, *p* = 0.071), and the nonlinear component was not significant (*χ^2^* = 0.31, df = 1, *p* = 0.576). With 0 months as the reference, the HR showed a decreasing trend as work duration increased. The HRs at 60, 120, and 180 months were 0.881 (95% CI 0.804–0.966), 0.815 (95% CI 0.732–0.908), and 0.775 (95% CI 0.666–0.902), respectively (Figure 1).

### 3.7 Sensitivity analysis: forcing age into the composite endpoint model

In the primary model for the composite endpoint, age was not included because it did not reach the prespecified threshold (*p* < 0.1) in univariable analysis. To further rule out residual confounding by age, we forced age into the multivariable model. Age showed no significant association with the composite endpoint (HR = 0.999, 95% CI 0.989–1.010, *p* = 0.874). The effect estimates of work duration, smoking, systolic blood pressure, and other core variables remained essentially unchanged (Supplementary Table S2).

### 3.8 Sensitivity analysis: binary blood pressure classification

To verify the robustness of the SBP effect, the variable was dichotomized (≥130 mmHg vs. <130 mmHg) and the multivariable Cox model was refitted. The high SBP group was associated with a 66.3% increased risk of the composite endpoint (HR = 1.663, 95% CI 1.327–2.085, *p* < 0.001). The effect directions of work duration categories, smoking, and other core variables were consistent with the primary model (Table 5).

**Table 5:** Sensitivity analysis: binary classification of systolic blood pressure (composite endpoint).

Variable	HR	95% CI	***p***
PTA (dB HL)	1.016	0.998–1.034	0.087
Platelet count (×10^9^/L)	1.001	1.000–1.002	0.093
SBP (mmHg)			
<130 mmHg	1.000	Reference	
≥130 mmHg	1.663	1.327–2.085	<0.001
Work duration			
0 months	1.000	Reference	
1–60 months	0.748	0.585–0.957	0.021
61–120 months	0.772	0.596–1.000	0.050
>120 months	0.603	0.453–0.803	0.001
Smoking			
No	1.000	Reference	
Yes	1.207	1.043–1.398	0.012
Sex			
Female	1.000	Reference	
Male	1.246	0.888–1.747	0.203

## 4 Discussion

### 4.1 Core findings and the healthy worker effect

This 3-year follow-up study of noise-exposed workers, using a composite cardiovascular endpoint (hypertension or electrocardiographic abnormality), identified smoking and elevated SBP as independent risk factors and demonstrated that the apparent protective effect of work duration is consistent with the HWE. The secondary endpoint analyses confirmed the robustness of the composite endpoint: smoking was significant in both the composite and electrocardiographic abnormality models, while SBP was significant in both the composite and hypertension models, with consistent effect directions. The binary sensitivity analysis further showed a 66.3% increased risk of the composite endpoint among those with baseline SBP ≥ 130 mmHg, indicating that intervention is warranted even at high-normal blood pressure levels.

The healthy worker effect was evaluated using three complementary approaches. First, restriction—excluding pre-employment workers—showed that the apparent protective effect persisted among active workers (HR = 0.787 among active workers, with 1–60 months as the reference). Second, restricted cubic spline analysis confirmed a monotonic decreasing trend in HR with increasing work duration (*P* for overall association = 0.071; *P* for nonlinearity = 0.576). Third, forcing age into the multivariable model did not materially change the effect estimates (Supplementary Table S2), ruling out age as a major confounder. Collectively, these findings are consistent with baseline health selection bias, a core manifestation of the HWE—workers who had been exposed to noise for many years yet maintained normal blood pressure and normal ECG at baseline were inherently healthy survivors. However, the observed inverse association may also reflect other selection mechanisms, such as the healthy worker survivor effect—whereby workers with declining health may transfer to less exposed jobs or leave the workforce entirely—as well as the screening effect of periodic occupational health examinations that remove workers with newly detected abnormalities from the active workforce (12, 13). Given that this was a fixed cohort with no loss to follow-up, the contribution of the healthy worker survivor effect during the study period is likely limited, but pre-baseline selection processes cannot be excluded. The HWE comprises the healthy worker hire effect and the healthy worker survivor effect; the latter, as a time-varying confounder, is difficult to fully adjust using conventional regression methods (10, 14).

### 4.2 Secondary endpoint analyses and implications

In the analysis with hypertension as the secondary endpoint, combined exposure to noise and dust (HR = 1.896) and to noise and chemical agents (HR = 1.580) significantly increased hypertension risk. This effect appeared only in the hypertension endpoint, suggesting synergistic pathways. Hai et al. also reported a higher hypertension risk from combined noise and dust exposure (OR = 2.69) than from single exposure (15). Mechanistically, noise induces oxidative stress and endothelial dysfunction through autonomic and HPA axis activation (16), while dust particulate exacerbates inflammation and vascular injury through the same pathways (17); chemical agents may similarly potentiate noise-induced cardiovascular harm (18). The shared oxidative stress and inflammatory cascades may represent a common pathway for these synergistic effects. Due to limited granularity in exposure classification, this study could not further quantify the interaction strength or statistical interaction type.

In the model with electrocardiographic abnormality as the endpoint, increasing age (HR = 0.984), work duration >120 months (HR = 0.728), and increasing BHFTA (HR = 0.983) all showed apparent protective effects. These findings suggest that baseline health selection bias also extends to the electrocardiographic abnormality endpoint. Older workers or those with longer work duration who had a normal baseline ECG were inherently healthy survivors, and their cardiac electrophysiological stability was thus greater. This pattern may also reflect collider bias, as restricting the cohort to workers with normal baseline ECG can create spurious inverse associations (13). Restricted cubic spline analyses showed monotonic decreasing trends with no evidence of nonlinearity for both age and BHFTA (nonlinear *p* = 0.353 and *p* = 0.466, respectively) (Supplementary Figures S2, S3), arguing against a biological protective mechanism. Collinearity diagnostics ruled out multicollinearity as an explanation for the divergent directions of PTA and BHFTA (all VIF < 2; Pearson *r* = 0.50). The apparent protective effect of BHFTA likely reflects the same baseline health selection bias: workers with elevated BHFTA had longer cumulative noise exposure and remained eligible for the cohort by virtue of their normal baseline ECG, and were thus inherently healthy survivors. In contrast, PTA primarily reflects low-frequency hearing, which is less sensitive to noise exposure and more directly associated with cardiovascular risk factors (19), and was therefore less affected by this selection process. Moreover, the 3-year follow-up may be too short to fully capture the causal chain from exposure to electrocardiographic abnormality. Although high-frequency hearing loss has been associated with arterial stiffness (20), its predictive value for short-term ECG changes remains uncertain. These findings await verification in larger cohorts with longer follow-up.

### 4.3 Implications

The findings of this study have clear implications for occupational health practice. Accurate identification of high-risk populations is fundamental to intervention: smokers, workers with SBP ≥ 130 mmHg, and those co-exposed to dust or chemical agents should be assigned the highest priority in cardiovascular risk management. A large cohort study by Suzuki et al. demonstrated that stage 1 hypertension (SBP 130–139 mmHg) according to the 2017 ACC/AHA guideline was associated with a 10% increased risk of composite cardiovascular events (21). In the present study, the same threshold corresponded to a 66.3% increased risk in this occupational population, strongly suggesting that high-normal blood pressure warrants dual monitoring and early intervention. Intervention strategies must shift from individual risk factor control to a multi-pathway exposure interaction network approach: in addition to noise reduction, smoking cessation, dynamic blood pressure management, and coordinated dust/chemical agent control should be integrated to interrupt the cascade of oxidative stress and inflammation. Policymakers and practitioners should remain vigilant about the HWE; the lower event rate among long-term active workers should not be misinterpreted as a protective effect of work duration, as this could weaken essential health surveillance.

### 4.4 Limitations

Several limitations should be acknowledged. First, although significant hazards of combined exposures were identified, the study did not quantify interaction strength or statistical interaction type. All workers met the L_eq, 8 h ≥ 80 dB(A) noise standard per workplace monitoring, but individual dosimetry data were unavailable, precluding stratification by exposure intensity. To assess the potential impact of unmeasured confounding from the lack of individual noise dosimetry data, E-value analyses were performed. For combined noise and dust exposure (HR = 1.896), the E-value was 3.20, suggesting that an unmeasured confounder would need to be associated with both exposure and outcome by a risk ratio of approximately 3.2 to fully explain the observed effect. For elevated SBP ≥ 130 mmHg (HR = 1.663), the E-value was 2.71. A semi-quantitative cumulative exposure index showed consistent results but was limited by subjective weighting (Supplementary Table S3). Future studies should collect individual-level cumulative exposure doses and apply multi-pollutant joint models. Second, the relatively short 3-year follow-up may limit the ability to detect longer-term cardiovascular effects of occupational noise exposure, and the paradoxical associations of hearing indicators require verification in cohorts with longer follow-up, incorporating job transfers and hearing protection use as covariates. Third, although the healthy worker effect was evaluated using restriction, restricted cubic spline analysis, and age-adjusted sensitivity analysis, full quantitative adjustment using G-estimation could not be performed because of categorical exposure and employment status variables and discrete-time outcome data (12, 13). Furthermore, the study design could not disentangle the healthy worker hire effect from the healthy worker survivor effect, nor could it fully exclude alternative explanations such as job rotation or the screening effect of health examinations. Fourth, this study was based on a single-center occupational health examination population with a small female sample, limiting generalizability to broader noise-exposed populations and female workers. Future research should integrate individual-level noise exposure doses, biomarkers of combined exposure, and long-term follow-up endpoint events within a unified framework to provide a more precise basis for cardiovascular risk assessment and intervention strategies in noise-exposed workers.

## Data Availability

The original contributions presented in the study are included in the article/Supplementary material, further inquiries can be directed to the corresponding author.

## References

[1] Zong X Hu H Li H Wang Y Du L Xu C . Global, regional, and national burden of DALYs attributable to occupational risks, 1990-2021: trends and projections to 2030. Soc Sci Med. (2026) 389:118810. doi: 10.1016/j.socscimed.2025.118810, 41313905

[2] Teixeira LR Pega F de Abreu W de Almeida MS de Andrade CAF Azevedo TM . The prevalence of occupational exposure to noise: a systematic review and meta-analysis from the WHO/ILO joint estimates of the work-related burden of disease and injury. Environ Int. (2021) 154:106380. doi: 10.1016/j.envint.2021.106380, 33875242 PMC8204275

[3] Zheng J Zhang S Wang H Yu Y Hu W. Surveillance of noise exposure level in the manufacturing industry—China, 2020. China CDC Wkly. (2021) 3:906–10. doi: 10.46234/ccdcw2021.222 34745689 PMC8563329

[4] Zhang C Wang J Wang H Zhang H. Surveillance of noise exposure level in industrial enterprises-Jiangsu Province, China, 2022. Front Public Health. (2024) 12:1230481. doi: 10.3389/fpubh.2024.1230481, 38410664 PMC10894969

[5] National Center for Cardiovascular Diseases, The Writing Committee of the Report on Cardiovascular Health and Diseases in China. Chinese cardiovascular health and disease report 2024: summary. Chin Circ J. (2025) 40:521–59. doi: 10.3969/j.issn.1000-3614.2025.06.001 (in Chinese) 39401992

[6] Münzel T Molitor M Kuntic M Hahad O Röösli M Engelmann N . Transportation noise pollution and cardiovascular health. Circ Res. (2024) 134:1113–35. doi: 10.1161/CIRCRESAHA.123.323584, 38662856

[7] Skogstad M Johannessen HA Tynes T Mehlum IS Nordby KC Lie A. Systematic review of the cardiovascular effects of occupational noise. Occup Med (Lond). (2016) 66:10–6. doi: 10.1093/occmed/kqv148, 26732793

[8] Yang Y Zhang E Zhang J Chen S Yu G Liu X . Relationship between occupational noise exposure and the risk factors of cardiovascular disease in China: a meta-analysis. Medicine (Baltimore). (2018) 97:e11720. doi: 10.1097/MD.0000000000011720, 30045338 PMC6078733

[9] Torén K Neitzel RL Eriksson HP Andersson E. Occupational exposure to noise and dust in Swedish soft paper mills and mortality from ischemic heart disease and ischemic stroke: a cohort study. Int Arch Occup Environ Health. (2023) 96:965–72. doi: 10.1007/s00420-023-01980-x, 37261594 PMC10361880

[10] Li CY Sung FC. A review of the healthy worker effect in occupational epidemiology. Occup Med (Lond). (1999) 49:225–9. doi: 10.1093/occmed/49.4.225, 10474913

[11] Park EJ Yuk K Jeong J Lee WJ. Confounding and the healthy worker survivor effect in studies of medical radiation workers: a systematic review of methodological approaches. Epidemiol Health. (2026) 48:e2026009. doi: 10.4178/epih.e2026009, 41643634 PMC13033441

[12] Neophytou AM Picciotto S Hart JE Garshick E Eisen EA Laden F. A structural approach to address the healthy-worker survivor effect in occupational cohorts: an application in the trucking industry cohort. Occup Environ Med. (2014) 71:442–7. doi: 10.1136/oemed-2013-102017, 24727736 PMC4051133

[13] Brown DM Picciotto S Costello S Neophytou AM Izano MA Ferguson JM . The healthy worker survivor effect: target parameters and target populations. Curr Environ Health Rep. (2017) 4:364–72. doi: 10.1007/s40572-017-0156-x, 28712046 PMC5693751

[14] Consonni D D’Errico A Merletti F. "Occupational epidemiology". In: Ahrens W Pigeot I, editors. Handbook of Epidemiology. New York: Springer New York (2025)

[15] Hai R Gao XP Xu LJ Liang XQ Liu MT Ning L. Effects of combined exposure to dust and noise on blood pressure and electrocardiogram of mechanical manufacturing workers. Zhonghua Lao Dong Wei Sheng Zhi Ye Bing Za Zhi. (2025) 43:275–80. doi: 10.3760/cma.j.cn121094-20240120-00030, 40328622

[16] Münzel T Schmidt FP Steven S Herzog J Daiber A Sørensen M. Environmental noise and the cardiovascular system. J Am Coll Cardiol. (2018) 71:688–97. doi: 10.1016/j.jacc.2017.12.015, 29420965

[17] Brook RD Rajagopalan S Pope CA 3rd Brook JR Bhatnagar A Diez-Roux AV . Particulate matter air pollution and cardiovascular disease: an update to the scientific statement from the American Heart Association. Circulation. (2010) 121:2331–78. doi: 10.1161/CIR.0b013e3181dbece1, 20458016

[18] Bayo Jimenez MT Hahad O Kuntic M Daiber A Münzel T. Noise, air, and heavy metal pollution as risk factors for endothelial dysfunction. Eur Cardiol. (2023) 18:e09. doi: 10.15420/ecr.2022.41, 37377448 PMC10291605

[19] Oron Y Elgart K Marom T Roth Y. Cardiovascular risk factors as causes for hearing impairment. Audiol Neurootol. (2014) 19:256–60. doi: 10.1159/000363215 25073427

[20] Wang D Xiao Y Li W Feng X Yi G Chen Z . Association of noise exposure, plasma microRNAs with arterial stiffness among Chinese workers. Environ Pollut. (2022) 311:120002. doi: 10.1016/j.envpol.2022.120002, 35995288

[21] Suzuki Y Kaneko H Okada A Fujiu K Takeda N Morita H . BP classification using the 2017 ACC/AHA BP guidelines with risk of cardiovascular events in older individuals. J Cardiol. (2024) 84:394–403. doi: 10.1016/j.jjcc.2024.07.005, 39067569

