# Supplementary material for: Cardiovascular composite events and the healthy worker effect among noise-exposed workers: a retrospective cohort study
Source: Front Public Health. 2026 Jul 7;14:1889187. doi: 10.3389/fpubh.2026.1889187 (PMC13385450; doi:10.3389/fpubh.2026.1889187)
Supplement: Supplementary file 1 [file Supplementary_file_1.docx]

Supplementary Material

# Supplementary Tables

| Table S1 Baseline characteristics of noise‑exposed workers and comparison between groups | | | | | | | | | | | |  |  |  |
| --- | --- | --- | --- | --- | --- | --- | --- | --- | --- | --- | --- | --- | --- | --- |
| **Variable** | | Overall （n=1381) | | | Non‑event （n=606) | | | Event （n=775) | | ***p*** | |  |  |  |
| Age (years) | | 32.4±8.0 | | | 32.8±8.0 | | | 32.2±8.0 | | 0.182 | |  |  |  |
| Systolic blood pressure  (mmHg) | | 113.1±10.6 | | | 111.9±9.8 | | | 113.9±11.1 | | ＜0.001 | |  |  |  |
| Diastolic blood pressure  (mmHg) | | 75.9±7.0 | | | 75.2±6.6 | | | 76.4±7.2 | | 0.002 | |  |  |  |
| Heart rate (beats/min) | | 73.7±6.0 | | | 73.5±5.7 | | | 73.8±6.2 | | 0.429 | |  |  |  |
| BHFTA (dB HL) | | 20.6±9.6 | | | 20.8±9.7 | | | 20.6±9.4 | | 0.698 | |  |  |  |
| PTA (dB HL) | | 16.9±3.7 | | | 16.6±3.8 | | | 17.0±3.7 | | 0.041 | |  |  |  |
| Hemoglobin  (g/L) | | 150.9±12.1 | | | 150.4±12.8 | | | 151.3±11.5 | | 0.170 | |  |  |  |
| White blood cell count  (×10⁹/L) | | 6.6±1.6 | | | 6.6±1.6 | | | 6.7±1.6 | | 0.232 | |  |  |  |
| Platelet count (×10⁹/L) | | 232.4±56.7 | | | 228.4±58.6 | | | 235.6±54.9 | | 0.020 | |  |  |  |
| Sex | |  | | |  | | |  | | 0.065 | |  |  |  |
| Female | | 83 (6.0%) | | | 45 (7.4%) | | | 38 (4.9%) | |  | |  |  |  |
| Male | | 1298 (94.0%) | | | 561 (92.6%) | | | 737 (95.1%) | |  | |  |  |  |
| Work duration | |  | | |  | | |  | | 0.005 | |  |  |  |
| 0 months | | 117 (8.5%) | | | 38 (6.3%) | | | 79 (10.2%) | |  | |  |  |  |
| 1-60 months | | 639 (46.3%) | | | 279 (46%) | | | 360 (46.5%) | |  | |  |  |  |
| 61-120 months | | 378 (27.4%) | | | 161 (26.6%) | | | 217 (28%) | |  | |  |  |  |
| >120 months | | 247 (17.9%) | | | 128 (21.1%) | | | 119 (15.4%) | |  | |  |  |  |
| Hazard factor category | |  | | |  | | |  | | 0.139 | |  |  |  |
| Noise only | | 401 (29%) | | | 176 (29%) | | | 225 (29%) | |  | |  |  |  |
| Noise + dust | | 433 (31.4%) | | | 207 (34.2%) | | | 226 (29.2%) | |  | |  |  |  |
| Noise + chemical agents | | 445 (32.2%) | | | 185 (30.5%) | | | 260 (33.5%) | |  | |  |  |  |
| Noise + heat/others | | 102 (7.4%) | | | 38 (6.3%) | | | 64 (8.3%) | |  | |  |  |  |
| Family history | |  | | |  | | |  | | 0.764 | |  |  |  |
| No | | 1237 (89.6%) | | | 545 (89.9%) | | | 692 (89.3%) | |  | |  |  |  |
| Yes | | 144 (10.4%) | | | 61 (10.1%) | | | 83 (10.7%) | |  | |  |  |  |
| Smoking | |  | | |  | | |  | | 0.004 | |  |  |  |
| No | | 793 (57.4%) | | | 375 (61.9%) | | | 418 (53.9%) | |  | |  |  |  |
| Yes | | 588 (42.6%) | | | 231 (38.1%) | | | 357 (46.1%) | |  | |  |  |  |
| Alcohol consumption | |  | | |  | | |  | | 0.852 | |  |  |  |
| No | | 814 (58.9%) | | | 355 (58.6%) | | | 459 (59.2%) | |  | |  |  |  |
| Yes | | 567 (41.1%) | | | 251 (41.4%) | | | 316 (40.8%) | |  | |  |  |  |
| Note: Continuous variables are presented as mean ± standard deviation; categorical variables as n (%). P values were derived from t‑test for continuous variables and chi‑square test for categorical variables. SBP: Systolic blood pressure; BHFTA: binaural high‑frequency average hearing threshold; PTA: binaural pure‑tone average (speech frequency). This supplementary table provides the detailed baseline characteristics and between‑group comparisons referenced in the main text. | | | | | | | | | | | |  |  |  |
| **Table S2** Multivariable Cox regression analysis for the composite endpoint (including age as a covariate) | | | | | | | | | | | | |  | |
| **Variable** | | | | **HR** | | **95% CI** | | | | | ***p*** | |  | |
| Age (years) | | | | 0.999 | | 0.989–1.010 | | | | | 0.874 | |  | |
| Platelet count (×10⁹/L) | | | | 1.001 | | 1.000–1.002 | | | | | 0.086 | |  | |
| PTA (dB HL) | | | | 1.016 | | 0.998–1.034 | | | | | 0.082 | |  | |
| SBP (mmHg) | | | | 1.013 | | 1.005–1.020 | | | | | <0.001 | |  | |
| Work duration | | | |  | |  | | | | |  | |  | |
| 0 months | | | | 1.000 | | Reference | | | | |  | |  | |
| 1–60months | | | | 0.784 | | 0.613–1.003 | | | | | 0.053 | |  | |
| 61–120months | | | | 0.790 | | 0.609–1.026 | | | | | 0.077 | |  | |
| >120months | | | | 0.627 | | 0.460–0.854 | | | | | 0.003 | |  | |
| Smoking | | | |  | |  | | | | |  | |  | |
| No | | | | 1.000 | | Reference | | | | |  | |  | |
| Yes | | | | 1.191 | | 1.030–1.379 | | | | | 0.019 | |  | |
| Sex | | | |  | |  | | | | |  | |  | |
| Female | | | | 1.000 | | Reference | | | | |  | |  | |
| Male | | | | 1.203 | | 0.853–1.697 | | | | | 0.291 | |  | |
| Note: The model included all variables listed in the table. This supplementary table presents the sensitivity analysis with age forced into the multivariable model, as referenced in the main text. | | | | | | | | | | | | |  | |
| **Table S3** Sensitivity analysis: semi-quantitative cumulative exposure index and the composite endpoint. | | | | | | | | | | | | | |  |
| **Variable** | **HR** | | | | | | **95% CI** | | ***p*** | | | | |  |
| Cumulative exposure (per unit) | 0.998 | | | | | | 0.997–0.999 | | 0.004 | | | | |  |
| Platelet count (×10⁹/L) | | | 1.001 | | | | 1.000–1.002 | | 0.073 | | | | |  |
| PTA (dB HL) | | | 1.017 | | | | 0.999–1.035 | | 0.065 | | | | |  |
| SBP (mmHg) | | | 1.013 | | | | 1.006–1.020 | | <0.001 | | | | |  |
| Smoking | | |  | | | |  | |  | | | | |  |
| No | | | 1.000 | | | | Reference | |  | | | | |  |
| Yes | | | 1.211 | | | | 1.047–1.400 | | 0.010 | | | | |  |
| Sex | | |  | | | |  | |  | | | | |  |
| Female | | | 1.000 | | | | Reference | |  | | | | |  |
| Male | | | 1.179 | | | | 0.841–1.654 | | 0.339 | | | | |  |
| Note: The cumulative exposure index was calculated as work duration (months) × exposure intensity weight (noise only = 1, noise + dust/chemical agents = 1.5, noise + heat/other = 1.2). The model included all variables listed in the table. | | | | | | | | | | | | | |  |

# Supplementary Figure


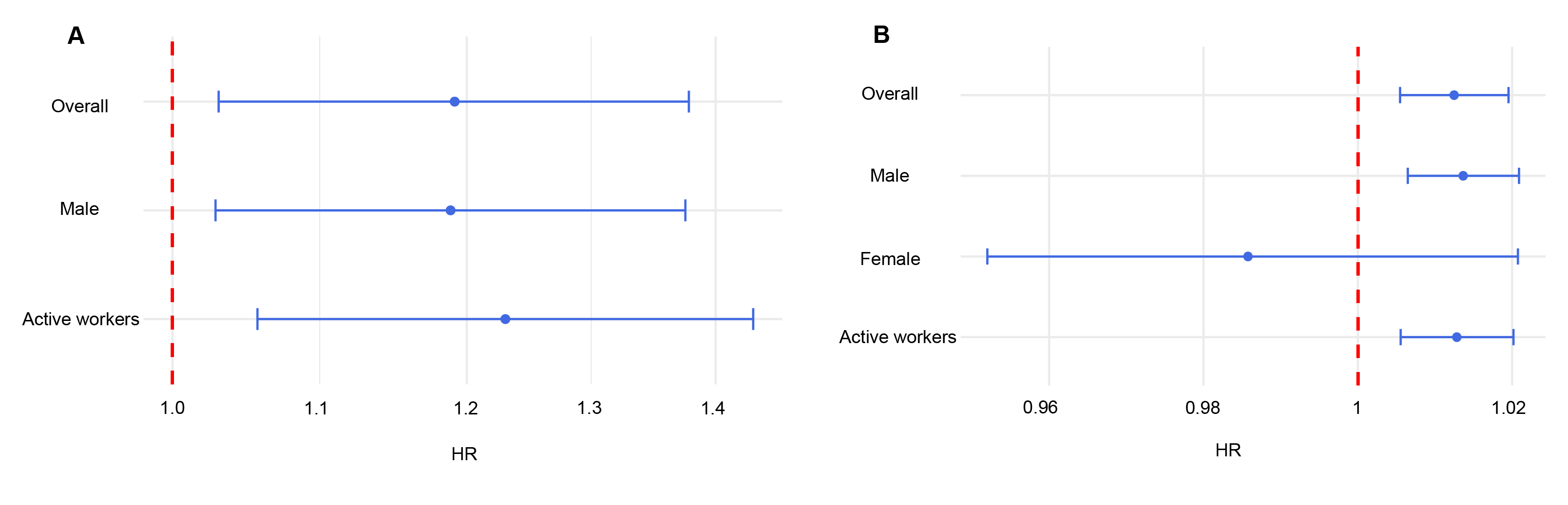


**Supplementary Figure S1** Subgroup analysis of the effects of smoking and systolic blood pressure on the composite endpoint. (A) Effect of smoking. (B) Effect of systolic blood pressure (per 1 mmHg increase). Hazard ratios (HRs) and 95% confidence intervals are shown for each subgroup. The red dashed line indicates HR = 1. The model was adjusted for work duration categories, platelet count, sex, and pure-tone average (PTA). The female subgroup (n = 83) was not included in (A) because the effect could not be estimated due to the small sample size; it was included in (B). The overall, male, and active worker subgroups showed consistent effect directions in both (A) and (B) (all *p* < 0.05).


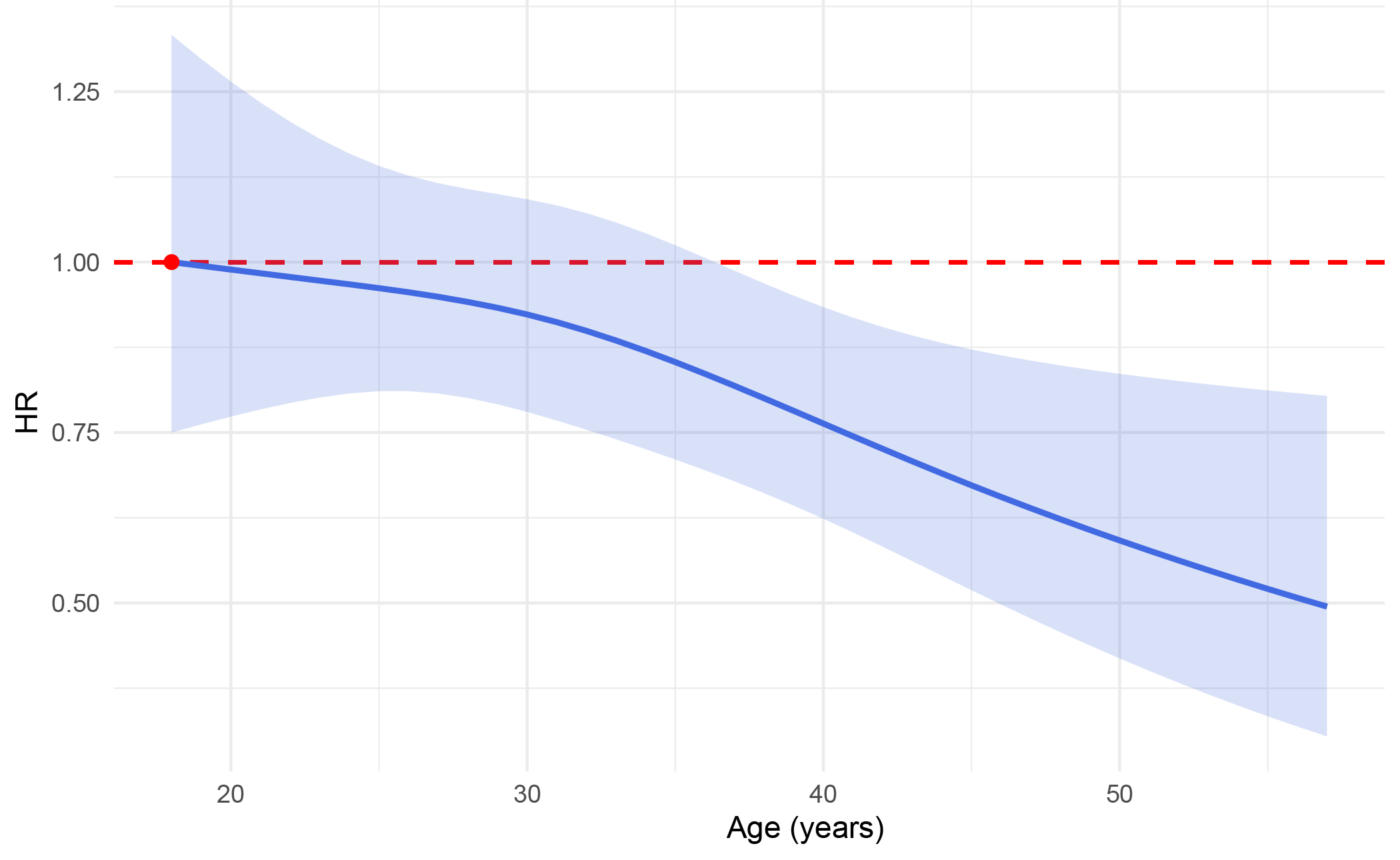


**Supplementary Figure S2** Dose-response relationship between age and electrocardiographic abnormality (restricted cubic spline). Knots were placed at the 10th, 50th, and 90th percentiles. The model was adjusted for work duration (1–60 months), smoking (no), PTA (17.0 dB HL), BHFTA (20.6 dB HL), hazard factor category (noise + chemical agents), and platelet count (228 × 10⁹/L). The solid line represents the hazard ratio (HR), the shaded area indicates the 95% confidence interval, and the red dashed line marks HR = 1.

**
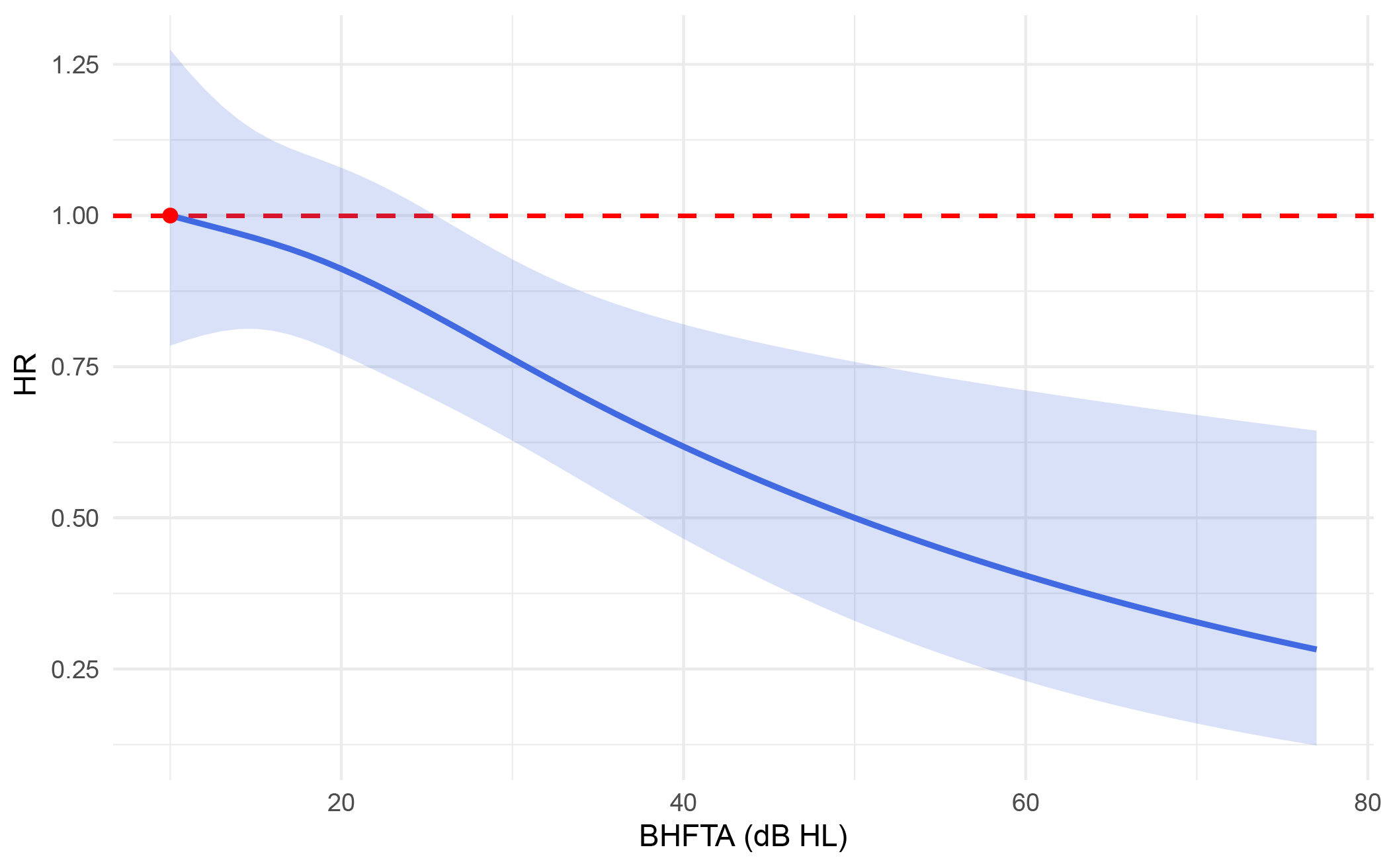
**

**Supplementary Figure S3** Dose-response relationship between BHFTA and electrocardiographic abnormality (restricted cubic spline). Knots were placed at the 10th, 50th, and 90th percentiles. The model was adjusted for age (32 years), work duration (1–60 months), smoking (no), PTA (17.0 dB HL), hazard factor category (noise + chemical agents), and platelet count (228 × 10⁹/L). The solid line represents the hazard ratio (HR), the shaded area indicates the 95% confidence interval, and the red dashed line marks HR = 1.
